# Effects of Replacing Fishmeal with the Mixture of Cottonseed Protein Concentrate and *Clostridium autoethanogenum* Protein on the Growth, Nutrient Utilization, Serum Biochemical Indices, Intestinal and Hepatopancreas Histology of Rainbow Trout (*Oncorhynchus mykiss*)

**DOI:** 10.3390/ani13050817

**Published:** 2023-02-23

**Authors:** Hongfei Huang, Xiaoqin Li, Kailin Cao, Xiangjun Leng

**Affiliations:** 1 National Demonstration Center for Experimental Fisheries Science Education, Shanghai Ocean University, Shanghai 201306, China; 2 Centre for Research on Environmental Ecology and Fish Nutrition (CREEFN) of the Ministry of Agriculture, Shanghai Ocean University, Shanghai 201306, China; 3 Shanghai Collaborative Innovation for Aquatic Animal Genetics and Breeding, Shanghai Ocean University, Shanghai 201306, China

**Keywords:** rainbow trout, cottonseed protein concentrate, *Clostridium autoethanogenum* protein, growth performance, nutrient utilization, serum biochemical indices, intestinal histology, hepatopancreas histology

## Abstract

**Simple Summary:**

As the price of fishmeal continues to rise, it is urgent to seek new protein sources to decrease fishmeal inclusion in aquafeeds. However, anti-nutrient factors limit the application of plant proteins in aquafeeds. Among alternative protein sources, cottonseed protein concentrate (CPC) and *Clostridium autoethanogenum* protein (CAP) contain are considered promising substitutes because of their few anti-nutrient factors and complementary effect in amino acid composition. In this study, we evaluated the effects of replacing fishmeal with a mixture of CPC and CAP (1:1) on growth performance, nutrient utilization, serum biochemical indices, and intestinal and hepatopancreas histology of rainbow trout (*Oncorhynchus mykiss*). Based on the results, the mixture of CPC and CAP could replace 50% of dietary fishmeal without negative effects on rainbow trout.

**Abstract:**

The purpose of this study was to develop the potential of cottonseed protein concentrate (CPC) and *Clostridium autoethanogenum* protein (CAP) in the diet of rainbow trout (*Oncorhynchus mykiss*) by evaluating the effects of substituting fishmeal with a CPC and CAP mixture on growth performance, nutrient utilization, serum biochemical indices, intestinal and hepatopancreas histology. In a basal diet containing 200 g/kg fishmeal (Con), the mixture of CPC and CAP (1:1) was used to reduce dietary fishmeal to 150, 100, 50 and 0 g/kg, to form five diets with the same crude protein and crude lipid contents (CON, FM-15, FM-10, FM-5 and FM-0). Then, the five diets were fed to rainbow trout (35.00 ± 0.05 g) for 8 weeks. The weight gain (WG) and feed conversion ratio (FCR) of the five groups were 258.72%, 258.82%, 249.90%, 242.89%, 236.57%, and 1.19, 1.20, 1.24, 1.28, 1.31, respectively. FM-5 and FM-0 groups showed significantly lower WG and higher FCR than the CON group (*p* < 0.05). In terms of whole-body composition, such as moisture, crude ash, and crude protein, no significant difference was observed among all the groups (*p* > 0.05), except that significantly higher crude lipid content was detected in the FM-0 group than in the CON group (*p* < 0.05). In the FM-5 and FM-0 groups, protein efficiency, protein retention, intestinal protease activity and amylase activity were significantly lower than in the CON group (*p* < 0.05). Compared to the CON group, the serum contents of glucose and total cholesterol in the FM-0 group as well as MDA in the FM-5 and FM-0 groups were significantly increased, and catalase, superoxide dismutase, and total antioxidant capacity were decreased (*p* < 0.05). In intestine and hepatopancreas histology, the intestinal villus height in the FM-5 and FM-0 groups and villus width in the FM-0 group were decreased significantly (*p* < 0.05), while no significant difference in hepatopancreas morphology was observed among all the groups except that some vacuolization was observed in the FM-0 group (*p* > 0.05). In summary, the mixture of CPC and CAP can effectively replace 100 g/kg fishmeal in a diet containing 200 g/kg fishmeal without adverse effects on the growth performance, nutrient utilization, serum biochemical, or intestinal and hepatopancreas histology of rainbow trout.

## 1. Introduction

In aquaculture, more than 50% of production costs are incurred by feed [1], and fishmeal is the primary source of protein due to its balanced amino acid composition, high crude protein content, and unknown growth factors [2,3]. Due to the shortage of marine resources and the growing demand for fishmeal in aquafeeds, fishmeal price has been rising continuously. Therefore, new protein sources must be sought to replace or decrease fishmeal inclusion in aquafeeds. Previous studies have shown that some plant protein sources can partly or completely replace dietary fishmeal, including soybean meal [4], cottonseed meal [5], peanut meal [6], canola protein isolate [7], and corn protein [8]. However, the anti-nutrient factors in plant proteins may adversely affect the performance of fish [9]. Therefore, exploring new plant protein sources with low anti-nutrient factors seems to be critical for their wide application in aquafeeds.

Cottonseed protein concentrate (CPC) is derived from shelled cottonseed after oil extraction, dephenolization, and low-temperature drying. Generally, the crude protein content of CPC can reach 60% to 70% with plenty of arginine, histidine, and phenylalanine, and the free gossypol content is very low [10]. CPC was reported to replace 385 g/kg fishmeal in a basal diet containing 700 g/kg fishmeal without adversely affecting the growth and flesh quality of largemouth bass (*Micropterus salmoides*) [10]. The CPC prepared from glandless seeds completely replaced dietary fishmeal (400 g/kg) without adverse impacts on growth performance and feed utilization of black sea bass (*Centropristis striata*) [11]. Moreover, CPC has also been reported to partially replace fishmeal in the diets of golden pompano (*Trachinotus ovatus*) [12], hybrid grouper (*Epinephelus fuscoguttatus* × *E. lanceolatus*) [1], and large yellow croaker (*Larimichthys crocea*) [13].

As a new type of single-cell protein, *Clostridium autoethanogenum* protein (CAP) is obtained by liquid fermentation of *Clostridium ethanolum* with CO and ammonia as carbon and nitrogen sources. After the centrifugation and drying, the CAP product contains high levels of crude protein (80%) with high digestibility, high lysine content, and fewer anti-nutrient factors, but the arginine level is relatively low [14]. The successful replacement of fishmeal with CAP has been reported in some aquaculture species. For example, no adverse effects on the growth performance, feed utilization, or intestinal histology were found in largemouth bass when CAP replaced 150 g/kg fishmeal in a basal diet with fishmeal inclusion of 350 g/kg [15]. Similar results have also been reported in obscure pufferfish (*Takifugu obscurus*) [16], large yellow croakers [17], Pacific white shrimp (*Litopenaeus vannamei*) [18,19], juvenile turbot (*Scophthalmus maximus* L.) [15], and black sea bream (*Acanthopagrus schlegelii*) [20].

Rainbow trout (*Oncorhynchus mykiss*) is a cold water fish, widely cultured throughout the world. Rainbow trout is a fast growing fish with tender flesh, no intermuscular spines, and plenty of DHA and EPA [21]. Among salmon and trout, rainbow trout production is the second largest after Atlantic salmon (*Salmo salar*) [22]. As a carnivorous fish, the commercial diet for rainbow trout usually contains a high level of fishmeal, thus, reducing the fishmeal inclusion in feed is of great significance to develop sustainable aquaculture of rainbow trout. To date, it has been reported that some plant protein sources can replace fishmeal in rainbow trout diets, such as enzymatical soybean meal [23], fermented soybean meal [24], corn protein concentrate [8], canola: pea [25]. Recently, Zhao et al. [26] reported that CPC successfully replaced 50% of dietary fishmeal without adverse impacts on growth performance or antioxidant capacity of rainbow trout. However, the control diet in that study contained a high level of fishmeal of up to 400 g/kg. Additionally, the inclusion of CAP in the diet has not yet been investigated in rainbow trout.

In terms of amino acid composition, CPC contains much arginine and little lysine and methionine, while CAP contains plenty of lysine and methionine and relatively low arginine levels. From this point of view, the two protein sources may have complementary effects in amino acid composition. Therefore, we evaluated the effects of substituting fishmeal with a mixture of CPC and CAP (1:1) on the growth performance, nutrient utilization, serum biochemical indices, and intestinal and hepatopancreas histology in the present study. The findings will develop the potential of CPC and CAP in carnivorous fish feeds and provide a low fishmeal diet for rainbow trout. 

## 2. Materials and Methods

### 2.1. Ethics Statement

All the procedures for handling animals involved in this experiment are in accordance with the regulations of the Experimental Animal Ethics Committee and the Institutional Animal Care Committee of Shanghai Ocean University (Approval code: SFI 2020-23 of 20 May 2020).

### 2.2. Experimental Diets and Design

First, a control diet was formulated to contain 200 g/kg fishmeal (CON). Then, the mixture of CPC and CAP (1:1) was used to reduce dietary fishmeal to 150, 100, 50 and 0 g/kg to form five diets with the same contents of crude protein (430 g/kg) and crude lipid (100 g/kg) (CON, FM-15, FM-10, FM-5 and FM-0). According to the method described by Xu et al. and Yao et al. [10,19], the protein ingredients such as fishmeal, CAP, CPC, etc., were ground and screened through a 60-mesh sieve, then mixed with liquid ingredients (fish oil, soybean oil, soybean lecithin). The five diets were prepared to form sinking pellets with a diameter of 3 mm using a single-screw extruder (LX-75 extruder, Longxiang Food Machinery Factory, Hebei, China). The extruding and air-drying temperatures were 90 ± 5 °C and 30 ± 2 °C, respectively. Then, all diets were stored at 4 °C until use. Table 1 shows the formulation and proximate composition of the diets.

CPC and CAP were provided by Chengrun Jinlan Biotechnology Co., Ltd. (Xinjiang, China) and Shoulang New Energy Technology Co., Ltd. (Tangshan City, China), respectively. The crude protein content of CPC and CAP was 615.1 g/kg and 842.1 g/kg, respectively, and the crude lipid content was 23.6 g/kg and 1.9 g/kg, respectively. The free gossypol content in CPC was 172.8 mg/kg. Fishmeal was steam-dried fishmeal (TASA), and the crude protein and lipid contents were 682.1 g/kg and 90.0 g/kg, respectively. Table 2 shows the proximate composition and amino acids profile of the four ingredients.

### 2.3. Management of Experimental Fish and Feeding

Rainbow trout were obtained from a local aquaculture farm in Sichuan (China). After transportation to the aquaculture station (Binhai, Shanghai, China), all fish were adapted to the environment and diets by feeding control diets for 2 weeks. A total of 300 juvenile rainbow trout (35.00 ± 0.05 g) were selected and randomly distributed into 15 indoor polyvinyl tanks with diameter, height, and water capacity of 1.0 m, 0.8 m, and 650 L, respectively. Three replicates (tanks) were designed for each treatment and each tank contained 20 fish. The system ran on the recirculation regime with a flowing rate of 10 L/min for each tank. During the feeding period, all the fish were fed at 09:00 and 16:00, and the feed intake was adjusted to ensure that no diet residue was found within 5 min when feeding was finished. The feces waste was siphoned out at the 2nd hour after feeding, and the cultured water was renewed (about 1/3) twice a week. During the feeding period of 56 days, the water quality was measured by measuring temperature (12–14 °C), dissolved oxygen content (6–8 mg/L), ammonia nitrogen content (<0.1 mg/L) and nitrite content (<0.1 mg/L).

### 2.4. Samples Collection

At the beginning of the feeding trial, ten fish were sampled and stored at −20 °C to determine the whole-body composition of initial fish. At the end of the feeding trial, all the fish were deprived of diets for 24 h, and then counted and bulk weighed for each individual tank to calculate weight gain (WG), feed conversion ratio (FCR), specific growth rate (SGR), survival, and feed intake (FI). Six fish per tank were randomly selected, of which three fish were used for the measurement of final fish whole-body composition. For the other three fish, body length and weight were measured to calculate condition factor (K), and then the blood was collected from the cordial vein with a syringe. After centrifuging at 3500 rpm for 10 min (4 °C), the supernatant was stored at −80 °C to determine serum biochemical indices. Then, the three fish were dissected, and the weight of visceral and hepatopancreas were measured to calculate the viscerosomatic index (VSI) and hepatosomatic index (HSI). Then, hepatopancreas (1 cm × 0.5 cm × 0.5 cm) and foregut (1 cm) tissues were stored in Bouin’s solution to observe tissue structure. The rest of the intestines were kept at −80 °C to determine digestive enzyme activity.

### 2.5. Measurement Indicators and Methods

#### 2.5.1. Growth Performance and Physical Indices

The growth performance and physical indices were calculated as follows:

Survival (%) = 100 × (final number of fish/initial number of fish)

WG (%) = 100 × [final weight (g) − initial weight (g)]/initial weight (g)

FCR = feed consumption (g)/weight gain (g)

SGR (%/day) = 100 × [ln final weight (g) − ln initial weight (g)]/days

K (g/cm^3^) = 100 × [body weight (g)/body length^3^ (cm)^3^]

VSI (%) = 100 × [visceral weight (g)/body weight (g)]

HSI (%) = 100 × [hepatopancreas weight (g)/body weight (g)]

FI (g/fish/d) = feed intake (g)/[(final fish number + initial fish number)/2]/days

#### 2.5.2. The Diets and Whole-Body Composition

AOAC method was used to measure moisture, crude ash, crude protein, and crude lipid contents in diets and whole-body composition [27]. To determine the moisture content, samples were dried in an oven at 105 °C to constant weight. The combustion method, the auto Kjeldahl system (2300 Auto analyzer, Foss Tecator, AB, Hoganas, Sweden), and the chloroform–methanol method were used to determine the contents of crude ash, crude protein, and crude lipid, respectively. The dietary amino acid composition was determined according to the description by Xu et al. [10]: The dried sample (70 mg) was added with 6 mol/L hydrochloric acid, then hydrolyzed at 110 °C for 24 h in a vacuum. After dilution (1:10), the amino acid content was determined by automatic analyzer (S-433D, Sykam, Munich, Germany).

#### 2.5.3. Serum Biochemical Indices

The serum activities of aspartate aminotransferase (AST), alanine aminotransferase (ALT), total antioxidant capacity (T-AOC), superoxide dismutase (SOD), catalase (CAT), and serum contents of malondialdehyde (MDA), triglyceride (TG), glucose (GLU), total cholesterol (TCHO), and total protein (TP) were determined by kits supplied by Nanjing Jiancheng Biological Co., Ltd., Nanjing, China.

#### 2.5.4. Nutrient Retention

The protein efficiency ratio (PER), protein retention (PR), and lipid retention (LR) were calculated as follows:

PER (%) = 100 × [final body weight (g) − initial body weight (g)]/protein intake (g)

PR (%) = 100 × [final body weight (g) × crude protein content of the final whole fish − initial body weight (g) × crude protein content of the initial whole fish]/protein intake (g)

LR (%) = 100 × [final body weight (g) × crude lipid content of the final whole fish − initial body weight (g) × crude lipid content of the initial whole fish]/lipid intake (g)

#### 2.5.5. Intestinal Digestive Enzyme Activity

After thawing at 4 °C, intestinal samples (0.1 g) were homogenized with nine times volume of ice-cold normal saline (1:9 *w*/*v*), and then centrifuged at 3000 rpm for 10 min at 4 °C. The supernatant was stored at −20 °C for digestive enzyme activity.

Protease activity was measured using the Folin phenol method [28], and the amount of enzyme that hydrolyzes casein and produces 1 μg tyrosine per minute (1 mg tissue protein) at pH 7.2 and 37 °C was defined as one unit (U/mgprot). Amylase activity was measured by iodine–starch colorimetry [29], and tissue protein (1 mg) reacted with the substrate at 37 °C for 30 min to hydrolyze 10 mg starch was defined as one unit (U/mgprot). The above indices were measured using kits supplied by Nanjing Jiancheng Biological Co., Ltd., Nanjing, China.

#### 2.5.6. Intestinal and Hepatopancreas Histology

For the embedding of foregut and hepatopancreas, we referred to the method described by Yang et al. [23]. A slicer (Leica RM2235, Heidelberg, Germany) was used to cut the sections (7 μm), which was then stained by hematoxylin-eosin. The microscope (Nikon YS100, Melville, NY, USA) was used to observe and photograph morphology of intestine and hepatopancreas, and then the villus height, villus width and muscle thickness were measured with image analysis software (Image J14.0), referring to the description of Zhao et al. [26].

### 2.6. Statistical Analysis

All data were presented as mean ± standard deviation (mean ± SD). Excel and SPSS 26.0 software were used to conduct a one-way ANOVA. Statistical significance among groups was determined using the Tukey’s multirange test if significant differences were detected.

## 3. Results

### 3.1. Growth Performance and Physical Indices

In Table 3, all the groups showed high survival, up to 100%. In the FM-15 and FM-10 groups, WG, FCR, and SGR were similar to those in the CON group (*p* > 0.05), while the FM-5 and FM-0 groups showed significantly lower WG and SGR (−6.12% and −3.51%, −8.56% and −4.82%), and higher FCR (+0.09, +0.12), than the CON group (*p* < 0.05). All the groups presented similar K, VSI, and HSI (*p >* 0.05).

### 3.2. Whole-Body Composition

The whole-body composition is shown in Table 4. The crude lipid content in the FM-0 group was significantly higher than in the CON group (*p* < 0.05). There were no significant differences in the content of moisture, crude ash, and crude protein among all the groups (*p* > 0.05). 

### 3.3. Nutrient Utilization and Intestinal Digestive Enzyme Activity

In Table 5, the increasing replacement of fishmeal with the mixture of CPC and CAP tended to decrease the protease and amylase activity, and PER. The protease activity in the FM-10, FM-5, and FM-0 groups and the amylase activity in the FM-5 and FM-0 groups was significantly lower than in the CON group (*p* < 0.05). The FM-5 and FM-0 groups also showed significantly lower PER and PR than the CON group (*p* < 0.05). The LR of the FM-5 group was significantly lower than that of the other groups (*p* < 0.05).

### 3.4. Serum Biochemical Indices

Table 6 shows the results of biochemical indices in serum. Serum contents of GLU and TCHO in the FM-0 group were significantly higher than those in the CON group (*p* < 0.05). Compared to the CON group, the FM-5 and FM-0 groups showed lower activities of SOD, T-AOC, and higher MDA content (*p* < 0.05). No significant difference in the activity of CAT, AST, ALT, or in the content of TG or TP was detected among all the groups (*p* > 0.05), but the increasing replacement of fishmeal with the CPC and CAP mixture tended to decrease the activity of CAT. 

### 3.5. Intestinal Morphology

**Figure 1:**
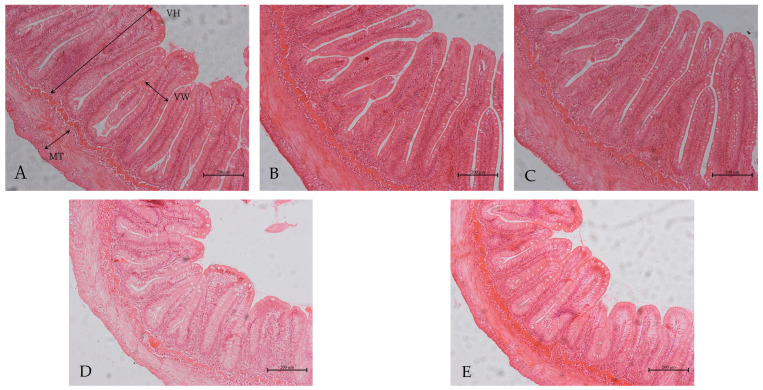
Effect of replacing fishmeal with cottonseed protein concentrate (CPC) and *Clostridium autoethanogenum* protein (CAP) mixture on foregut morphology of rainbow trout (10×). (**A**): CON; (**B**): FM-15; (**C**): FM-10; (**D**): FM-5; (**E**): FM-0; VH: villus height; VW: villus width; MT: muscle thickness. Scale bar = 200 μm.

Table 7 and Figure 1 depict the intestinal morphology. The villus height and muscle thickness in the FM-5 and FM-0 groups, as well as villus width in the FM-0 group, were significantly smaller than in the CON group (*p* < 0.05).

### 3.6. Hepatopancreas Morphology

**Figure 2:**
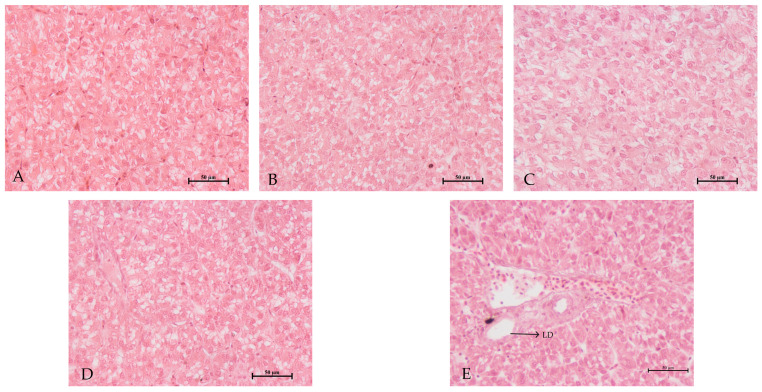
Effect of replacing fishmeal with CPC and CAP mixture on hepatopancreas morphology of rainbow trout (40×). (**A**): CON; (**B**): FM-15; (**C**): FM-10; (**D**): FM-5; (**E**): FM-0; LD: lipid droplet. Scale bar = 50μm.

As shown in Figure 2, vacuolation and lipid droplets were observed in some hepatocytes in the FM-0 group, while the nucleus and cell structure of hepatocytes in the other groups showed no abnormality.

## 4. Discussion

### 4.1. Growth Performance, Whole-Body Composition and Nutrient Utilization

The high replacement of fishmeal with plant protein sources may have adverse effects on the performance of aquatic animals [8]. In basal diets with fishmeal inclusion of 250 g/kg, 450 g/kg, 452 g/kg, and 400 g/kg, fermented soybean meal [24], solvent-extracted cottonseed meal [30], corn protein [8], and CPC [26] could reduce dietary fishmeal content to 150 g/kg, 225 g/kg, 338.1 g/kg, and 200 g/kg, respectively, without affecting the growth performance of rainbow trout. In the present study, the fishmeal content in the basal diet was only 200 g/kg, and the replaced fishmeal reached 100 g/kg. Both were much lower than the reported values in the above studies, realizing a low fishmeal diet for rainbow trout feed. It indicates that the CPC and CAP mixture is an excellent plant protein source to replace fishmeal in aquafeeds.

However, the WG and PR of rainbow trout were significantly decreased, and FCR was significantly increased, when dietary fishmeal was reduced to 50 g/kg by inclusion of the plant mixture (Table 3). In largemouth bass, the WG and feed utilization were significantly decreased when CPC was used to substitute 70% of fishmeal in the basal diet containing 700 g/kg fishmeal [10]. In Pacific white shrimp, the WG and PER were also decreased by the substitution of 45% of fishmeal with CPC in a diet containing 250 g/kg fishmeal [31]. Similarly, the WG of largemouth bass was significantly decreased when dietary fishmeal was reduced from 350 g/kg to 150 g/kg by CAP inclusion [15]. The substitution of 45% of dietary fishmeal with CAP also significantly decreased the WG and PER of Pacific white shrimp [19]. There are several reasons connected with the decreased growth and nutrient utilization by the excessive replacement of fishmeal with the CPC and CAP mixture in the present study: (1) The mixture contains a lower level of methionine than fishmeal (Table 2), and the high substitution of fishmeal may lead to deficiency of methionine. Methionine is a precursor of protein synthesis, which can be converted into taurine to promote fish growth [32,33]. (2) The high level of arginine in CPC may be another factor affecting the growth of rainbow trout. As excessive arginine causes antagonism with lysine, a high arginine level in the diet may negatively affect the lysine utilization and the growth of fish [34]. (3) Fishmeal contains high levels of taurine, hydroxyproline, and some unknown growth factors that are essential for growth and intestinal health, while bacterial and plant proteins lack these active compounds [15,19]. mTOR is a key signal molecule that regulates growth [35], and the efficiency of activating the mTOR signal pathway by fishmeal was found to be significantly higher than that by other protein sources such as CPC [36], corn gluten meal [35], and CAP [14,37]. Taurine is a conditionally essential amino acid in fish, which can promote the growth of fish [38]. The dietary supplementation of taurine reduced fishmeal inclusion from 240 g/kg to 160 g/kg without negative effects on the growth performance of largemouth bass [39]. In a low fishmeal diet (70 g/kg), the supplementation of taurine and selenium yeast increased the growth and protein utilization of black sea bass [40]. Hydroxyproline is a semi-essential amino acid in aquatic animals, which is a characteristic amino acid in collagen. In a turbot diet with high plant protein source inclusion, the supplementation of hydroxyproline (6 g/kg) increased the replaced ratio of fishmeal from 40% to 60% [41]. Aksnes et al. [42] also reported that the supplementation of hydroxyproline (2.9 g/kg) in a high plant protein source diet increased the WG of Atlantic salmon (*Salmo salar* L.) by 14%. (4) CAP and CPC have lower digestibility than fishmeal. The high fiber content in CPC and the presence of the cell wall in CAP may be an important factor affecting nutrient digestibility [43,44,45]. Li et al. [46] reported that the apparent digestibility coefficient of dry matter and crude protein of CPC were lower (−11.75% and −8.23%) than fishmeal in Pacific white shrimp, and high inclusion levels of CAP may reduce digestibility in animals.

The present findings showed no significant difference in whole-body composition such as moisture, crude protein, and crude ash when CPC and CAP mixture was used to substitution fishmeal. Similar results were also reported in rainbow trout [26] and black sea bream [20]. However, the FM-0 group presented significantly higher crude lipid content than the CON group. Liu et al. [47] found that the substitution of 75% of dietary fishmeal with CPC significantly increased the whole-body content of crude lipid in largemouth bass. Similar results were also described in southern flounder (*Paralichthys lethostigma*) [48]. Due to the decrease in feed utilization (including protein utilization), the intake of protein can not be effectively utilized to synthesize body protein, but rather is converted into lipid for deposition.

### 4.2. Intestinal Morphology and Digestive Enzyme Activity

The primary organ for digesting and absorbing nutrients of fish is the intestine, which impacts normal growth and development [49]. Intestinal protease and amylase are the main digestive enzymes in the intestine, and the villus height and villus width determine the contact area between mucosal epithelial cells and chyme, while muscle thickness is beneficial to intestinal peristalsis and chyme propulsion [50]. In the study, the intestinal protease and amylase activity tended to decrease with the decreasing inclusion of fishmeal, and the digestive enzyme activity, villus height, and muscle thickness in the FM-5 and FM-0 groups were significantly lower than those in the CON group. Wu et al. [51] found that the replacement of 45% of dietary fishmeal with CAP significantly decreased intestinal protease and amylase activity, resulting in intestinal damage and inflammation in large yellow croaker. In Pacific white shrimp, the intestinal villus height was significantly decreased when 40% of dietary fishmeal was replaced with CAP, but the supplementation of phosphorus improved the intestinal health [52]. Similar results were also observed in silver sillago (*Sillago sihama* Forsskál) [53] and large yellow croaker [13] when CPC replaced a high proportion of fishmeal. With the decrease in fishmeal inclusion, the bioactive substances such as taurine and trimethylamine oxide tend to decrease, which may reduce the ability of the pancreas and intestinal glands to secrete digestive enzymes. In juvenile cobia (*Rachycentron canadum*) [54] and golden pompano [55], the supplementation of taurine in the diet was reported to increase the trypsin activity and improve the intestinal structure, respectively.

### 4.3. Serum Biochemical Indices

AST and ALT are two important transaminases in amino acid metabolism, which exist in the heart and liver. When the liver is damaged, the cell membrane permeability of hepatocytes increases, and the two transaminases in the cytoplasm will be released into the blood, resulting in increased activity in the blood [56]. No significant difference in serum activities of AST and ALT was observed in the present study, although the hepatocytes in the FM-0 group presented some vacuolation and lipid droplets, indicating lipid accumulation in the liver. Such a result was consistent with the increasing whole-body crude lipid content in this group. However, in hybrid grouper [57] and largemouth bass [37], AST and ALT activity in serum were significantly increased when CPC and CAP replaced 36% and 50% of dietary fishmeal, respectively. It could be that the combination of CAP and CPC in the present study produced complementary effects and overcame their respective shortcomings. In addition, CPC is rich in arginine, and arginine could protect the liver by reducing the production of pro-inflammatory cytokines and free radicals [58].

Physiologically, blood GLU reflects the health of fish, which was also measured as a stress marker to evaluate the stress response caused by dietary changes [59]. The present study showed that blood GLU content was significantly increased only in the FM-0 group. In grass carp (*Ctenopharyngodon idllus*), the serum content of GLU was also significantly increased when 100 g/kg CAP was used to replace soybean meal [60]. In general, a high blood GLU level would lead to a stress response and affect the growth performance of fish [61]. Serum TCHO is an important indicator of body lipid metabolism. The content of TCHO in the FM-0 group was also significantly higher than that in the CON group, which was consistent with the increase in whole-body crude lipid content in this group.

Generally, the production and scavenging of active oxides are in dynamic equilibrium, and excessive free radicals lead to lipid peroxidation [62]. MDA is the toxic product of lipid peroxidation, and it can adversely affect health. SOD is the primary substance for organisms to scavenge oxygen free radicals, and CAT scavenge hydrogen peroxide to protect cells from toxicity, while T-AOC is usually used to evaluate the antioxidant capacity of animals [49,50,63,64]. In the present study, the activities of SOD and T-AOC in the FM-5 and FM-0 groups were all significantly lower, while the MDA content was significantly higher than those in the CON group, indicating that the high substitution of fishmeal by CPC and CAP mixture decreased the antioxidant capacity and broke the dynamic balance of free radicals. It has been reported that the high substitution of fishmeal (36%, 57.1%) with either CPC or CAP significantly increases the serum content of MDA in hybrid grouper [57] and largemouth bass [15]. Dietary supplementation of taurine alleviated the oxidative damage, such as decreasing the content of MDA [65], which has been confirmed in rice field eel (*Monopterus albus*) [66] and spotted knifejaw (*Oplegnathus punctatus*) [67]. 

In addition, the free gossypol in CPC may adversely affect the antioxidant system, although the free gossypol level in CPC is much lower than that in cottonseed meal. Gossypol can easily bind to proteins in the electron transfer chain of mitochondria, interfere with the function of mitochondria, and lead to excessive production of reactive oxygen species, thereby inhibiting the activity of various enzymes and causing oxidative damage [68]. 

## 5. Conclusions

In the present study, the mixture inclusion of CPC and CAP successfully decreased dietary fishmeal from 200 g/kg to 100 g/kg without adverse effects on the growth performance, nutrient utilization, serum biochemical, or intestinal and hepatopancreas histology of rainbow trout.

## Figures and Tables

**Table 1:** The diet formulation and proximate composition (air-dry basis, g/kg).

Ingredients ^1^	CON	FM-15	FM-10	FM-5	FM-0
Fishmeal	200.0	150.0	100.0	50.0	0.0
*Clostridium autoethanogenum* protein (CAP)	0.0	24.0	47.0	71.0	94.0
Cottonseed protein concentrate (CPC)	0.0	24.0	47.0	71.0	94.0
Soy bean meal	70.0	70.0	70.0	70.0	70.0
Soy protein concentrate	120.0	120.0	120.0	120.0	120.0
Wheat gluten	30.0	30.0	30.0	30.0	30.0
Wheat flour	270.0	268.0	268.0	266.0	266.0
Corn gluten meal	80.0	80.0	80.0	80.0	80.0
Meat meal	80.0	80.0	80.0	80.0	80.0
Beer yeast	50.0	50.0	50.0	50.0	50.0
Fish oil	20.0	24.0	28.0	32.0	36.0
Soybean oil	20.0	20.0	20.0	20.0	20.0
Soybean lecithin	20.0	20.0	20.0	20.0	20.0
Ca(H_2_PO_4_)_2_	20.0	20.0	20.0	20.0	20.0
Vitamin premix ^2^	10.0	10.0	10.0	10.0	10.0
Mineral premix ^3^	10.0	10.0	10.0	10.0	10.0
Total	1000.0	1000.0	1000.0	1000.0	1000.0
Proximate composition (g/kg)					
Moisture	63.0	61.1	59.5	71.7	73.7
Crude protein	438.0	439.5	435.7	429.0	430.1
Crude lipid	77.9	74.9	77.1	82.7	83.7
Crude ash	111.6	105.0	98.0	95.5	90.4

^1^ The protein contents of soy bean meal, soy protein concentrate, wheat flour, corn gluten meal, and meat meal were 426.7 g/kg, 648.9 g/kg, 145.5 g/kg, 588.6 g/kg, and 650 g/kg, respectively. ^2^ Vitamin premix (mg or IU/kg diet): vitamin A, 1000 IU; vitamin D_3_, 3000 IU; vitamin E, 150 IU; vitamin K_3_, 12.17 mg; vitamin B_1_, 20 mg; vitamin B_2_, 20 mg; vitamin B_3_, 100 mg; vitamin B_6_, 22 mg; vitamin B_12_, 0.15 mg; vitamin C, 300 mg; biotin, 0.6 mg; inositol, 400 mg; and folic acid, 8 mg. ^3^ Mineral premix (mg/kg diet): I, 1.5 mg; Mn, 11.45 mg; Co, 0.6 mg; Cu, 3 mg; Zn, 89 mg; Se, 0.24 mg; Mg, 180 mg; and Fe, 63 mg.

**Table 2:** The proximate composition and amino acid profile of the four ingredients (g/kg).

Items	CAP	CPC	CPC + CAP (1:1)	FM
Proximate composition (air-dry basis)				
Crude protein	842.1	615.1	728.6	682.1
Crude lipid	1.9	23.6	12.8	90.0
Crude ash	32.7	62.0	47.4	149.5
Moisture	71.4	52.5	62.0	68.0
Essential amino acids (dry-matter basis)				
Lysine	87.0	24.7	55.9	52.1
Methionine	22.9	8.5	15.7	20.3
Arginine	34.0	78.9	56.5	40.9
Histidine	16.8	18.0	17.4	20.7
Isoleucine	52.8	18.9	35.9	27.5
Leucine	63.8	34.4	49.1	52.6
Phenylalanine	33.0	35.3	34.2	35.9
Threonine	40.2	19.0	29.6	28.7
Tryptophan	6.2	8.1	7.2	7.0
Valine	54.4	26.6	40.5	33.7
Non-essential amino acids (dry-matter basis)				
Aspartic acid	95.4	56.6	76.0	61.0
Serine	32.1	26.5	29.3	26.1
Glutamic acid	97.8	123.7	110.8	87.5
Glycine	38.7	25.0	31.9	41.3
Alanine	46.3	23.6	35.0	44.2
Cysteine	7.1	9.5	8.3	4.1
Proline	24.0	21.7	22.9	28.4
Tyrosine	31.4	20.2	25.8	22.9
Total amino acids	783.9	579.2	681.6	634.9

**Table 3:** Effect of replacing fishmeal with cottonseed protein concentrate (CPC) and *Clostridium autoethanogenum* protein CAP mixture on growth performance of rainbow trout.

Items	CON	FM-15	FM-10	FM-5	FM-0
IBW (g)	35.05 ± 0.05	34.97 ± 0.06	35.00 ± 0.05	34.95 ± 0.05	35.02 ± 0.03
FBW (g)	125.73 ± 0.46 ^a^	125.47 ± 2.16 ^a^	122.47 ± 1.77 ^ab^	119.93 ± 0.88 ^b^	117.8 ± 1.41 ^b^
WG (%)	258.72 ± 1.42 ^a^	258.82 ± 5.75 ^a^	249.90 ± 5.03 ^ab^	242.89 ± 2.18 ^bc^	236.57 ± 4.04 ^c^
FCR	1.19 ± 0.01 ^c^	1.20 ± 0.03 ^c^	1.24 ± 0.02 ^bc^	1.28 ± 0.01 ^ab^	1.31 ± 0.02 ^a^
FI (g/fish/day)	1.93	1.93	1.93	1.93	1.93
SGR (% BW/day)	2.28 ± 0.01 ^a^	2.28 ± 0.03 ^a^	2.24 ± 0.03 ^ab^	2.2 ± 0.01 ^bc^	2.17 ± 0.02 ^c^
Survival (%)	100	100	100	100	100
K (g/cm^3^)	1.63 ± 0.01	1.55 ± 0.1	1.61 ± 0.08	1.64 ± 0.12	1.55 ± 0.01
VSI (%)	11.00 ± 0.50	10.13 ± 0.75	10.89 ± 0.99	10.42 ± 0.59	9.87 ± 0.7
HSI (%)	1.26 ± 0.09	1.23 ± 0.13	1.37 ± 0.13	1.28 ± 0.06	1.31 ± 0.02

In the same row, values with different small letter superscripts mean significant difference (*p* < 0.05). IBW: initial body weight; FBW: final body weight; WG: weight gain; FCR: feed conversion ratio; SGR: specific growth rate; K: condition factor; VSI: viscerosomatic index; HSI: hepatosomatic index.

**Table 4:** Effect of replacing fishmeal with CPC and CAP mixture on whole-body composition of rainbow trout (fresh weight, g/kg).

Items	CON	FM-15	FM-10	FM-5	FM-0
Moisture	708.5 ± 3.7	713.4 ± 15.7	714.8 ± 20.1	706.4 ± 15.7	700.2 ± 19.2
Crude ash	22.4 ± 1.6	22.7 ± 0.2	20.7 ± 0.2	21.0 ± 0.4	22.0 ± 1.7
Crude lipid	64.9 ± 2.7 ^b^	63.2 ± 2.5 ^b^	61.2 ± 5.0 ^b^	64.0 ± 3.9 ^b^	71.1 ± 1.0 ^a^
Crude protein	180.4 ± 1.6	181.5 ± 2.1	179.5 ± 6.4	178.4 ± 3.2	181.0 ± 1.8

In the same row, values with different small letter superscripts mean significant difference (*p* < 0.05). The same as below.

**Table 5:** Effect of replacing fishmeal with CPC and CAP mixture on nutrient utilization and intestinal digestive enzymes of rainbow trout.

Items	CON	FM-15	FM-10	FM-5	FM-0
Protease (U/mg prot)	16.10 ± 0.75 ^a^	14.26 ± 4.90 ^ab^	10.32 ± 0.67 ^bc^	8.16 ± 0.71 ^cd^	5.12 ± 0.21 ^d^
Amylase (U/mg prot)	0.70 ± 0.02 ^a^	0.60 ± 0.01 ^ab^	0.62 ± 0.03 ^ab^	0.44 ± 0.08 ^bc^	0.34 ± 0.02 ^c^
PER	1.91 ± 0.01 ^a^	1.90 ± 0.04 ^ab^	1.85 ± 0.04 ^abc^	1.83 ± 0.01 ^bc^	1.78 ± 0.02 ^c^
PR (%)	41.02 ± 0.18 ^a^	41.08 ± 0.81 ^a^	39.76 ± 0.67 ^ab^	39.12 ± 0.24 ^b^	38.99 ± 0.45 ^b^
LR (%)	80.42 ± 4.02 ^a^	81.00 ± 4.49 ^a^	77.35 ± 2.46 ^a^	68.43 ± 2.31 ^b^	77.59 ± 1.59 ^a^

In the same row, values with different small letter superscripts mean significant difference (*p* < 0.05). PER: protein efficiency ratio; PR: protein retention; LR: lipid retention.

**Table 6:** Effect of replacing fishmeal with CPC and CAP mixture on serum biochemical indices of rainbow trout.

Items	CON	FM-15	FM-10	FM-5	FM-0
AST (U/mL)	1.13 ± 0.07	1.09 ± 0.16	1.12 ± 0.19	1.17 ± 0.11	1.39 ± 0.26
ALT (U/mL)	6.78 ± 0.54	6.16 ± 0.73	6.40 ± 0.54	7.11 ± 0.09	7.40 ± 0.66
TG (mmol/L)	2.55 ± 0.07	2.56 ± 0.07	2.42 ± 0.12	2.68 ± 0.06	2.44 ± 0.05
GLU (mmol/L)	4.67 ± 0.34 ^b^	4.84 ± 0.28 ^ab^	4.58 ± 0.39 ^b^	5.09 ± 0.17 ^ab^	5.47 ± 0.23 ^a^
TCHO (mmol/L)	7.11 ± 0.48 ^b^	6.70 ± 0.25 ^b^	7.01 ± 0.11 ^b^	7.31 ± 0.33 ^b^	8.32 ± 0.22 ^a^
TP (g/L)	27.41 ± 2.11	28.48 ± 1.26	27.41 ± 2.41	26.56 ± 2.00	29.35 ± 0.84
MDA (nmol/mL)	14.03 ± 0.29 ^b^	14.68 ± 0.88 ^ab^	14.46 ± 1.15 ^ab^	16.45 ± 0.91 ^a^	16.29 ± 1.19 ^a^
CAT (U/mL)	4.24 ± 0.92	3.73 ± 0.50	3.72 ± 0.37	3.17 ± 0.61	2.76 ± 0.64
SOD (U/mL)	49.93 ± 1.38 ^a^	50.78 ± 5.18 ^a^	50.57 ± 2.55 ^a^	40.49 ± 1.04 ^b^	42.16 ± 1.46 ^b^
T-AOC (mmol/L)	0.79 ± 0.04 ^a^	0.78 ± 0.05 ^a^	0.80 ± 0.05 ^a^	0.67 ± 0.03 ^b^	0.68 ± 0.01 ^b^

In the same row, values with different small letter superscripts mean significant difference (*p* < 0.05). AST: aspartate aminotransferase; ALT: alanine aminotransferase; TG: triglyceride; GLU: glucose; TCHO: total cholesterol; TP: total protein; MDA: malondialdehyde; CAT: catalase; SOD: superoxide dismutase; T-AOC: total antioxidant capacity.

**Table 7:** Effect of replacing fishmeal with the CPC and CAP mixture on the foregut morphology of rainbow trout.

Items	CON	FM-15	FM-10	FM-5	FM-0
Villus height (μm)	734.9 ± 27.0 ^a^	737.2 ± 39.2 ^a^	764.4 ± 37.8 ^a^	670.4 ± 32.8 ^b^	603.1 ± 58.4 ^c^
Villus width (μm)	161.9 ± 17.6 ^a^	165.6 ± 4.9 ^a^	168.4 ± 11.7 ^a^	167.0 ± 23.5 ^a^	127.6 ± 9.0 ^b^
Muscle thickness (μm)	140.6 ± 5.8 ^a^	141.1 ± 6.4 ^a^	139.2 ± 20.1 ^a^	120.0 ± 9.3 ^b^	119.4 ± 6.0 ^b^

In the same row, values with different small letter superscripts mean significant difference (*p* < 0.05).

## Data Availability

All dates are contained within the article.
